# Genomic Regions Associated with IgE Levels against *Culicoides* spp. Antigens in Three Horse Breeds

**DOI:** 10.3390/genes10080597

**Published:** 2019-08-08

**Authors:** Liesbeth François, Hanne Hoskens, Brandon D. Velie, Anneleen Stinckens, Susanne Tinel, Chris Lamberigts, Liesbet Peeters, Huub F. J. Savelkoul, Edwin Tijhaar, Gabriella Lindgren, Steven Janssens, Bart J. Ducro, Nadine Buys, Anouk Schurink

**Affiliations:** 1 Livestock Genetics, Department of Biosystems, KU Leuven, B-3001 Leuven, Belgium; 2 Department of Human Genetics, KU Leuven, B-3000 Leuven, Belgium; 3 School of Life & Environmental Sciences, B19-603 University of Sydney, Sydney, NSW 2006, Australia; 4 Research Group Livestock Physiology, Department of Biosystems, KU Leuven, Leuven, B-3001 Leuven, Belgium; 5 Biomedical Research Institute, Hasselt University, B-3590 Diepenbeek, Belgium; 6 Cell Biology and Immunology Group, Wageningen University & Research, 6700 AH Wageningen, The Netherlands; 7 Department of Animal Breeding and Genetics, Swedish University of Agricultural Sciences, 75007 Uppsala, Sweden; 8 Animal Breeding and Genomics, Wageningen University & Research, 6700 AH Wageningen, The Netherlands; 9 Centre for Genetic Resources, The Netherlands (CGN), Wageningen University & Research, 6700 AH Wageningen, The Netherlands

**Keywords:** Belgian Warmblood horse, diagnostic ELISA test, genome-wide association study, Icelandic horse, IgE, insect bite hypersensitivity, Shetland pony, summer eczema

## Abstract

Insect bite hypersensitivity (IBH), which is a cutaneous allergic reaction to antigens from *Culicoides* spp., is the most prevalent skin disorder in horses. Misdiagnosis is possible, as IBH is usually diagnosed based on clinical signs. Our study is the first to employ IgE levels against several recombinant *Culicoides* spp. allergens as an objective, independent, and quantitative phenotype to improve the power to detect genetic variants that underlie IBH. Genotypes of 200 Shetland ponies, 127 Icelandic horses, and 223 Belgian Warmblood horses were analyzed while using a mixed model approach. No single-nucleotide polymorphism (SNP) passed the Bonferroni corrected significance threshold, but several regions were identified within and across breeds, which confirmed previously identified regions of interest and, in addition, identifying new regions of interest. Allergen-specific IgE levels are a continuous and objective phenotype that allow for more powerful analyses when compared to a case-control set-up, as more significant associations were obtained. However, the use of a higher density array seems necessary to fully employ the use of IgE levels as a phenotype. While these results still require validation in a large independent dataset, the use of allergen-specific IgE levels showed value as an objective and continuous phenotype that can deepen our understanding of the biology underlying IBH.

## 1. Introduction

The most common skin disease in horses, insect bite hypersensitivity (IBH), is the result of a cutaneous allergic reaction to salivary antigens from *Culicoides* spp. [1,2,3]. The affected horses show hair loss, excoriation, crusting, scaling, and thickening of the skin [2]. Not only does this lead to a substantial reduction in welfare of the affected horse, but also the commercial value is reduced and there is an increased monetary and time cost to the owner to alleviate the signs [2]. IBH prevalence ranges between 8.1% in Swedish-born Icelandic horses [4], 8.8% in Shetland ponies [5], and 10% in Belgian Warmblood horses [6]. Studies suggest that the high prevalence in Icelandic horses imported from Iceland (50% or higher) can be the result of their non-exposure to *Culicoides* spp. early in life, due to the absence of these midges in this country [7,8]. Icelandic horses that were imported as weanlings from Iceland to areas in Europe, where they were exposed to *Culicoides* spp., do not show an increased sensitivity when compared to European Icelandic horses, which is suggestive of the development of immune tolerance at a young age [9].

The genetic background of IBH has been confirmed in several studies with a heritability that ranged from 0.08 to 0.36 in different breeds [4,5,6,10,11,12]. Until now, genomic research has not yet been able to identify the genetic variants that cause IBH, although several regions and variants of interest have been found. For instance, the involvement of the major histocompatibility complex (MHC) class II region has been confirmed in several breeds [10,13,14,15,16,17,18].

IBH mainly involves a Type I hypersensitivity reaction that is mediated by the presence of allergen-specific Immunoglobulin E (IgE) antibodies. After initial sensitization to the allergen (being *Culicoides* spp. in case of IBH), subsequent exposure to the allergen results in the degranulation of mast cells and the release of various mediators (e.g., histamine) through the crosslinking of the allergen specific IgE bound to high-affinity IgE receptors on these mast cells. This degranulation causes vascular permeability, which leads to e.g., swelling and itchiness [19]. As IBH is a reaction of the immune system to antigens of *Culicoides* spp., an indirect enzyme-linked immunosorbent assay (ELISA) test that measures these allergen-specific IgE levels can be used to assess the horse’s sensitivity. The test is based on the observation that IBH affected horses present higher titers of allergen-specific IgE antibodies [20,21,22,23,24,25,26,27,28]. Recombinant antigens that are derived from different *Culicoides* spp. and whole body extracts (WBE) are employed. An ELISA test that is based on *C. nubeculosus* allergens has been applied in Belgian Warmblood horses, while an ELISA test using allergens from *C. obsoletus* has been employed in Icelandic horses, Shetland ponies, and Belgian Warmblood horses [26,29,30,31,32]. While IBH is most commonly identified through the scoring of the clinical signs, misdiagnosis is possible, and the use of a diagnostic test could provide a more objective phenotype [4,5,18,33]. Contrary to the normal case-control design that is used in most genome-wide association (GWA) studies for IBH, the use of observed IgE levels as an objective, independent, and quantitative phenotype could improve the power to detect causal genetic variants [34]. Van der Meide and colleagues [26] showed that there is variation in the IgE levels within cases and within controls, and the IgE levels and severity of signs were correlated [30]. Provided that this variation is related to the sensitivity to IBH, the IgE levels will be more accurate in representing this sensitivity when compared to a case-control classification.

In our study, we used the observed IgE levels against several *Culicoides* spp. antigens in Belgian Warmblood horses, Icelandic horses, and Shetland ponies as a quantitative phenotype to perform a GWA study. We combined independent immunological and genetic research on IBH, both being performed by two different research groups on different breeds. This resulted in a unique and first study using a quantitative phenotype in the context of GWA studies, which aimed at increasing our understanding of the underlying biology of IBH, possibly improving the therapy the efficiency of breeding programs to decrease IBH prevalence.

## 2. Materials and Methods

Several aspects differed between the breed as we combined independent immunological and genetic research on IBH from two different research groups on different breeds: selection criteria to sample cases and controls, allergens that are used to determine the IgE levels, and genotyping arrays to genotype the horses. These differences should be taken into account when interpreting the potential differences in the results between the breeds.

### 2.1. Sampling

One research group studied Icelandic horses (*n* = 127) and Shetland ponies (*n* = 200). Individuals from both breeds were sampled in areas with different risk status for IBH in the Netherlands. Individuals were sampled while using a matched case-control design to avoid population stratification, as described by Schurink and colleagues [14,15]. All the individuals were inspected by one of the researchers and a veterinarian, while using a standardized scoring system and questionnaire to obtain detailed information regarding the history and management of IBH (for more details, see Schurink and colleagues [15]). Cases were defined as ponies showing mild to severe clinical signs, and controls were free of signs despite exposure to *Culicoides* spp. Therefore, controls were required to be at least four years of age (related to the average age of onset of IBH being between two and four years of age). Additionally, at the same premises, a case had to be present, to ensure the exposure to *Culicoides* spp. Paternal half-sibs were sought, to match cases and controls on genealogy as well. Most cases and controls were present for two or more years on their current premises to ensure constant management practices (>90%). The Shetland pony population consisted of 200 ponies (103 cases and 97 controls), while the Icelandic horse populations contained 127 horses (56 cases and 71 controls). 

Belgian Warmblood horses (*n* = 223) were studied by the other research group and sampled during stable visits and competitions in the summer, as described by Peeters and colleagues [29]. Horse owners were asked about various environmental traits (e.g., soil and stable type) and horse traits (e.g., IBH status and body condition) by a trained investigator while using a questionnaire [33]. The cases were defined as horses showing or that had shown clinical signs. If the owner had never observed sings since the ownership of the horse began, the horse was classified as control. Additionally, controls were required to be at least 3 years of age and kept on premises with a case. Horses were selected for genotyping while using the exclusion protocol that is presented in Appendix A—Figure A1, leading to the selection of 107 controls and 116 cases. In short, a horse was excluded when IBH status was unknown or unclear and when it was not a purebred Belgian Warmblood horse. In selecting cases and controls, pedigree was considered (no full-sibs allowed), and an equal distribution between cases and controls across several traits was aimed for.

Information regarding the date of sampling, age, and sex was available in all breeds, information on coat color and size category was available for Shetland ponies, information on coat color and import status was available for the Icelandic horses, and information on vegetation, humidity, body condition, fitness, stable management, training frequency, and deworming frequency was available for Belgian Warmblood horses [14,15,29].

Concerning the Shetland ponies and Icelandic horses, the Board on Animal Ethics and Experiments from Wageningen University approved blood sample collection (experiments 2009055 and 2010109). For the Belgian Warmblood horses, the Ethical Committee for Animal Experiments of the Katholieke Universiteit Leuven approved blood sample collection (approval number P061-2012, date of approval 18 April 2012).

### 2.2. Phenotype–IgE Levels Determined with ELISA Test

Diagnostic ELISA tests were performed to determine the IgE levels (OD_450nm_ values) against *Culicoides obsoletus* (all breeds) and *Culicoides nubeculosus* (only Belgian Warmblood horses), as described by Peeters and colleagues [29], Schaffartzik and colleagues [25], and van der Meide and colleagues [26]. Belgian Warmblood horses were sampled from June through September to ensure exposure to *Culicoides* spp. Shetland ponies and Icelandic horses were sampled in September and October. More information regarding the recombinant allergens that were used in the different populations can be found in Table 1. The highest sensitivity (93.2%) in Shetland ponies and Icelandic horses was obtained while using a *C. obsoletus* whole body extract (WBE) [26]. In Belgian Warmblood horses, the highest sensitivity (70%) was obtained while combining IgE levels against Culn4, Culo1 (Genbank id JX512273), and Culo2 (Genbank id JX512274) with *C. obsoletus* WBE [29]. The obtained specificity of the ELISA test in the Belgian Warmblood horses using these allergens was 97% [29].

### 2.3. DNA Extractions, Genotyping, and Quality Control

For the Icelandic and Shetland samples, DNA was extracted, as described by Schurink and colleagues [14], and the samples were genotyped while using the Illumina equine HD chip containing 65,157 single-nucleotide polymorphisms (SNPs). For the Belgian Warmblood samples, DNA was extracted from blood samples (200 µL) while using the Qiagen kit. Belgian Warmblood samples were genotyped using the Affymetrix Axiom Equine Genotyping Array containing 670,796 SNPs. Quality control was separately assessed in each population using GenABEL in R [35]. SNPs with a minor allele frequency (MAF) <1% and call-rate <95% were discarded and a threshold for minimum call rate per horse was set to 90%. In the Icelandic horse population, 127 horses and 52,655 SNPs (80.8% of the SNPs) remained after quality control, while 199 ponies and 49,567 SNPs (76.1% of the SNPs) remained in the Shetland pony population. In the Belgian Warmblood horse population, 214 horses and 474,128 SNPs (70.7% of the SNPs) retained after quality control. 

### 2.4. Genome-Wide Association (GWA) Study

IgE levels (OD_450nm_ values) against *Culicoides* spp. were log transformed while using the natural logarithm, as preliminary analyses showed the presence of heterogeneous variance and deviations from a normal distribution (Appendix B—Table A1). These log transformed IgE levels were used as continuous traits in the GWA analyses. IgE levels against each allergen were separately tested, which resulted in 11 GWA studies in Belgian Warmblood horses and 10 GWA studies in both Icelandic horses as well as Shetland ponies.

In the Belgian Warmblood horse population, several covariates were obtained from the questionnaire that was performed at each sampling [33]: sex, age (in years), ageClass (categorical variable), period of sampling, date of sampling, vegetation at pasture, humidity around pasture, type of stabling, weight of the horse, fitness level of the horse, number of trainings per week, and number of parasite treatments per year. All of these variables were tested for having a significant effect on the IgE levels. While several variables were found to explain part of the variation observed in different IgE levels, only the variable “ageClass” had a significant effect on all IgE levels (except Culn1). Therefore, only this variable was included in the model in the downstream analyses. In the Shetland pony population, age, size category, coat color, month of sampling, and location of sampling were tested as potential factors or covariates having a significant effect on IgE levels. The age of the pony had a significant effect on several *C. obsoletus* specific IgE levels in the Shetland pony population, and therefore included in the model. In the Icelandic horse population, sex and age were tested as potential factors or covariates having a significant effect on IgE levels. However, none of these variables had a significant effect on IgE levels. Due to differences in the age of exposure to *Culicoides* spp., unequal sampling of cases and controls of imported Icelandic horses (in total 19 horses), and the subsequent confounding effect, only European born Icelandic horses were used to perform the GWA study (*n* = 127). The significance of factors and covariates was determined while using a linear model (command *anova(lm,...)* in the nnet package) in R software [38].

**Figure 1:**
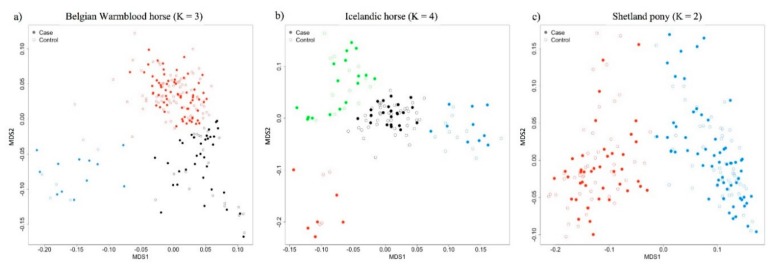
Multidimensional scaling (MDS) plot with the number of clusters assigned in (**a**) Belgian Warmblood horses (K = 3), (**b**) Icelandic horses (K = 4), and (**c**) the Shetland ponies (K = 2). Additionally, to investigate whether the higher density SNP-array in Belgian Warmblood horses rendered more significant results, a subset of SNPs was obtained representing the overlap between the 65 k Illumina equine HD chip and the 670 k Affymetrix Axiom Equine Genotyping Array. As a case study, a GWA analysis was performed in the Belgian Warmblood horse population using all SNPs (474,128 after quality control) and using the subset of SNPs for L_Culn7 (as most significant results were obtained using the IgE levels against this *C. nubeculosus* allergen).

For each population, the genomic kinship matrix was computed and standard K-means clustering was performed to determine the number of potential subpopulations [39]. In the Belgian Warmblood horse population K = 3 was established, in the Shetland pony population K = 2, and K = 4 in the Icelandic horse population. Clustering within the populations did not coincide with the case-control status (Figure 1). Additionally, no outliers were apparent in any of the studied populations while using the multidimensional scaling (MDS) plot (Figure 1). GWA analyses were performed with the IgE levels against *Culicoides* antigens as a continuous trait using the GenABEL package in R [35,38]. To avoid spurious associations that may arise with unusual allele frequency differences between subpopulations [40], a mixed model-structured association approach (“*mmscore*” function) was employed with a correction for substructure through the use of the assigned cluster as a covariate [35]. The *p*-values were corrected for inflation factor lambda (Pc1df). The conservative Bonferroni corrected significance level was 1.01 × 10^−6^ in Shetland ponies, 9.50 × 10^−7^ in Icelandic horses, and 1.05 × 10^−7^ in Belgian Warmblood horses (being *α*/*n*, where *α* is the desired significance level (0.05) and *n* is the number of SNPs that was tested). As none of the SNPs passed Bonferroni corrected significance threshold, the 50 SNPs that were most significantly associated with antigen-specific IgE levels per breed were presented.

Additionally, to investigate whether the GWA analysis using IgE levels against *Culicoides* spp. is indeed more powerful in detecting associated genetic variants as compared to a case-control GWA analysis, a case-control GWA analysis was performed in the Belgian Warmblood horse population using a structured association analysis (“*qtscore*”) in the GenABEL package in R [35,38]. A case-control GWA study using the same Shetland ponies and Icelandic horses as in our study was already performed and presented in scientific literature [14,15]. The results from our GWA analyses using the IgE levels will be compared to the results that were obtained in these case-control studies [14,15].

## 3. Results

**Figure 2:**
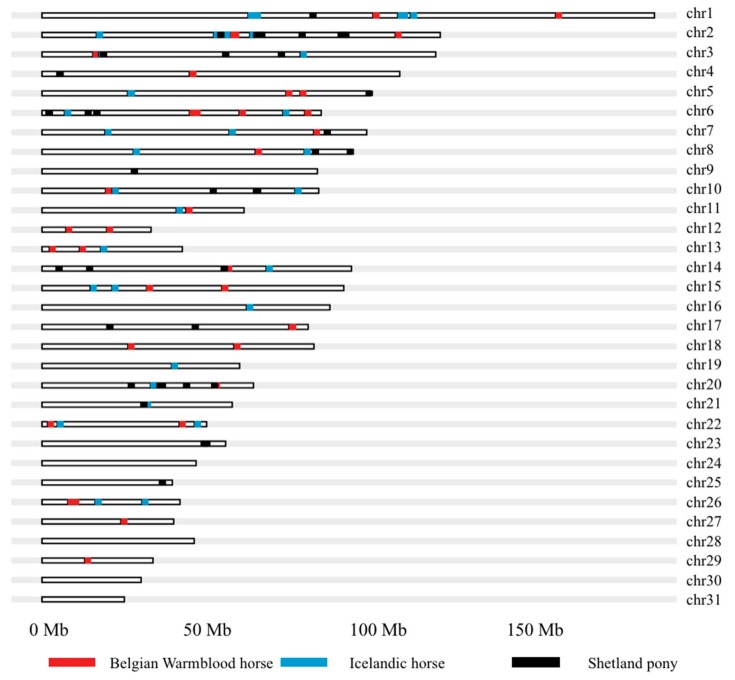
Karyogram from the genome-wide association (GWA) analyses indicating the location of 50 SNPs per breed most significantly associated with IgE levels against *Culicoides* spp. allergens in Belgian Warmblood horses, Icelandic horses, and Shetland ponies.

The locations of the 50 SNPs per breed most significantly associated with IgE levels against *Culicoides* spp. allergens in Belgian Warmblood horses, Icelandic horses, and Shetland ponies are presented in Figure 2. Genomic regions that are associated with IgE levels against *Culicoides* spp. allergens across two or all breeds were found on ECA2:52–65 Mb, ECA3:71–78 Mb, ECA6:1–15 Mb, ECA7:82–85 Mb, ECA15:14–31 Mb, and ECA20:26–52 Mb (Figure 2, Appendix C—Table A2). Per breed, various regions were identified containing several SNPs that were associated with IgE levels against *Culicoides* spp. allergens. For Belgian Warmblood horses, these regions were ECA4:44 Mb, ECA5:74–78 Mb, ECA6:44–46 Mb, ECA11:43 Mb, ECA17:74–75 Mb, and ECA22:41 Mb (Figure 2, Appendix C—Table A2). For Icelandic horses, these regions were ECA1:62–64 Mb, ECA1:107–111 Mb, ECA2:52–63 Mb, ECA3:78 Mb, and ECA15:14–21 Mb. For the Shetland ponies, these regions were ECA4:4 Mb, ECA6:13–15 Mb, ECA7:85 Mb, ECA20:26–51 Mb, ECA21:30 Mb, and ECA23:48–49 Mb.

Information on the *p*-value of these SNPs, allele frequency, and allele substitution effect of the minor allele, as well as which *Culicoides* spp. allergen it was associated with, are presented in Table A2 (Appendix C). Some *Culicoides* spp. allergens obtained more significant results (Appendix C—Table A2), where, in Shetland ponies, 11 out of 50 SNPs were associated with L_Culo4, 11 SNPs with L_Culo6, and 17 SNPs with L_Culo7. In Icelandic horses, 13 out of 50 SNPs were associated with L_Culo4. In Belgian Warmblood horses, 13 out of 50 SNPs were associated with L_WBE and nine SNPs with L_Culn7.

The GWA analysis in the Belgian Warmblood horse population using a subset of SNPs representing the density in the two other populations rendered less significant results as compared to the GWA analysis using all SNPs (474,128 after quality control; Appendix D—Figure A2a,b).

**Figure A3:**
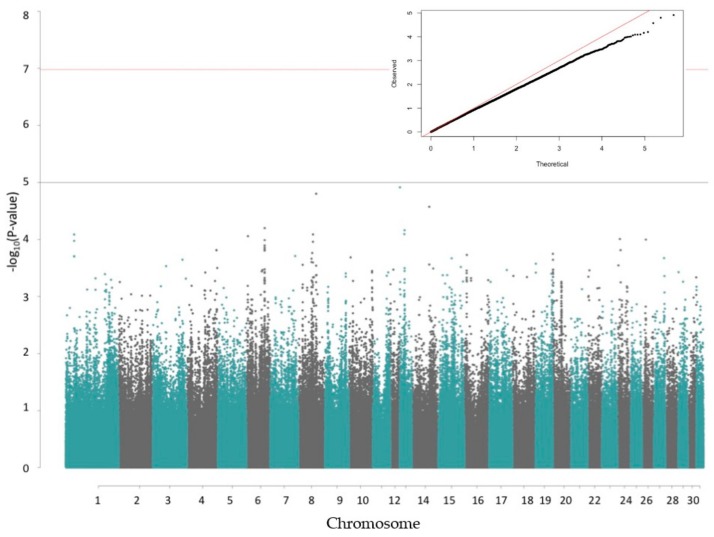
Manhattan plot with position on the genome on the x-axis and significance (−log10(*p*-values)) on the y-axis in the Belgian Warmblood horse population for IBH status (case-control) using a structured association analysis in GenABEL package in R [35,38]. The red line represents the Bonferonni corrected significance threshold and the grey line a nominal significance threshold (*p*-value = 1 × 10^−5^). Q-Q plot indicating that inflation factor lambda was <1, requiring no further adjustment.

The results from the case-control GWA analysis in the Belgian Warmblood horse population are presented in Figure A3 (Appendix E). The case-control GWA analysis obtained less significant results when compared to the GWA analysis using IgE levels against *Culicoides* spp. allergens (Appendix C—Table A2 and Appendix D—Figure A2). *P*-value of the SNP most significantly associated with case-control status in the Belgian Warmblood horse population was 1.22 × 10^−5^ (AX-103321942 on ECA13:728,323 bp).

A comprehensive list of genomic regions that were associated in any of the three investigated breeds can be found in Table A3 (Appendix F). Previously identified associations from case-control GWA analyses (in any equine breed) in proximity to the genomic regions identified in our study are also presented.

## 4. Discussions

IBH is a serious welfare concern in several horse breeds, and several studies have tried to identify the genetic background of IBH in order to gain a better understanding of the disease and optimize selection strategies [14,15,16,17,18]. The misclassification of IBH can occur as clinical signs are not always visible, depending on exposure to *Culicoides* spp., and it may originate from some other cause, making a typical case-control GWA study less powerful [41,42,43] when compared to a continuous, independent, and objective trait that reflects the underlying sensitivity. Our study used a diagnostic ELISA test with high sensitivity and specificity to assess the IgE levels against *Culicoides* spp. antigens, resulting in an objective, independent, and quantitative phenotype as input for a GWA study [34,44]. The classical diagnosis of IBH combines the history of the horse and the observation of clinical signs that follow a seasonal pattern [2,3,45]. As this might lead to the failure to detect affected horses, a diagnostic test could give a more reliable observation of the horse’s sensitivity to *Culicoides* spp. if they have been exposed to the midges. Several diagnostic tests are available, such as the measurement of inflammatory mediators [46,47], the measurement of wheal size after intradermal allergen challenge [24,48], and the use of an ELISA test to analyze allergen-specific IgE levels in the serum of horses [25,26,29]. These tests mostly use recombinant allergens from different species of *Culicoides*, and they were able to differentiate between cases and controls in several breeds. While using a *C. obsoletus* WBE, a sensitivity of 93.2% in Shetland ponies and Icelandic horses was reached with a specificity of 90% [26]. In Belgian Warmblood horses a sensitivity of 70% and a specificity of 97% was obtained combining Culn4, Culo1, and Culo2 with IgE levels against *C. obsoletus* WBE [29]. In contrast to the classical IBH case-control scoring system, the IgE levels from the allergen-specific ELISA test provided a continuous and objective phenotype that allowed for a more optimal and powerful GWA analysis [34,45]. When compared to the case-control GWA analysis that was performed in our study, more significant associations were identified when using IgE levels. Van der Meide and colleagues [26] showed that there is variation in IgE levels within cases and within controls, and IgE levels against *Culicoides* spp. antigens were correlated with the severity of IBH clinical signs [30]. Similarly, in humans, a clear correlation has been established between the total IgE levels or allergen-specific IgE levels and severity of eczema signs [49]. Provided that the variation in IgE levels against *Culicoides* spp. is related to IBH sensitivity, IgE levels will be more accurate in representing this sensitivity when compared to a case-control classification. Moreover, through using IgE levels from an ELISA test with high specificity and sensitivity misdiagnosis is limited and a more direct genotype to phenotype relationship is established.

Associations with IgE levels were found on most chromosomes, where across-breed associations were identified on ECA2, ECA3, ECA6, ECA7, ECA15, and ECA20. The complex nature of IBH supports the selection of any SNP surpassing a reasonable significance threshold, even though no SNP reached genome-wide significance based on the strict Bonferroni correction [50,51,52,53]. Although a common genetic background is to be expected across different breeds concerning a Th2 driven IgE production and induction of allergic diseases, selection criteria to sample cases and controls, allergens used to determine the IgE levels, and the SNP density differed between the three breeds under study, which might have contributed to a few regions being associated across breeds. Even with the use of the same density chip in the Shetland pony and Icelandic horse, certain regions in the genome might contain different SNP densities, as each breed underwent a separate quality control. In addition, linkage disequilibrium (LD) between SNPs and a causal mutation might differ between the breeds [54].

When comparing the different GWA analyses on IBH using a case-control approach that was based on clinical signs [14,15,16,17,18] and our quantitative trait being antigen-specific IgE levels against *Culicoides* spp. revealed several associated genomic regions across various chromosomes in common (Appendix F). For instance, the identified region on ECA6 in Belgian Warmblood horses (44–46 Mb) was in close proximity to regions that were significantly associated with IBH in Shetland ponies [15] and Icelandic horses [17]. Other regions that were associated with IgE levels and IBH status across two or more unrelated breeds were found on ECA1:100 Mb, ECA2:105–107 Mb, ECA4:43–44 Mb, ECA8:62–64 Mb and 78–79 Mb, ECA9:26–27 Mb, ECA10:19–21 Mb and 49–51 Mb, ECA11:40–43 Mb, ECA15:31–33 Mb, ECA16:62–65 Mb, ECA17:74–77 Mb, ECA20:24–26 Mb, 29–35 Mb and 49–52 Mb, and ECA26:14–16 Mb. Besides the MHC class I, II, and III region on ECA20 containing genes that have critical functions in immunity (extensively discussed by [17]), no obvious candidate genes that are involved in the Th2 driven IgE production and induction of allergic disease were detected in close proximity to the identified regions significantly associated with allergen-specific IgE levels or previous GWA analyses based on case-control status. Although several previously identified genomic regions that are associated with IBH were confirmed, new regions of interest were also found. These new regions might potentially represent additional or other pathways identified due to the use of the more objective and quantitative IgE levels, as compared to case-control status representing the presence or absence of clinical signs.

The GWA analyses in Belgian Warmblood horses resulted in more significant associations when IgE levels were used as compared to a case-control status. These analyses allowed for a direct comparison, as the same horses were used. The identified associations based on case-control status in the same Shetland ponies as investigated in our study were less significant [15]. However, similar significance levels as in our study were observed in Exmoor ponies and Friesians horses [17,18] while using the same SNP density as in Belgian Warmblood horses. The Exmoor pony and Friesian horse populations represent inbred horses breeds and larger samples were used [17,18] as compared to our breeds. Inbreeding likely resulted in higher LD [55] and, when combined with the larger sample size, more power to detect causal variants. A comparison with results from Shrestha and colleagues [16] and Schurink and colleagues [14] is quite challenging, as a Bayesian approach was employed, presenting the percentage of genetic variance explained by 1 Mb windows. The use of allergen-specific IgE levels showed value as a continuous and objective phenotype.

The ELISA test applied in Belgian Warmblood horses used a different set of *Culicoides* antigens (several recombinant allergens of *C. nubeculosus* and *C. obsoletus*) as compared to the test applied in Shetland ponies and Icelandic horses where only allergens of *C. obsoletus* were used [26,29,30,31,32]. Anderson and colleagues [56] showed that cases reacted to all the extracts of *Culicoides* spp., even when cases had not been exposed to most of the species, which suggested that the allergen(s) were present in all the investigated extracts (native *C. obsoletus*, *C. cockerellii*, *C. imicola*, *C. biguttatus*, *C. variipennis,* and non-native *C. obsoletus*). However, using the diagnostic test in Belgian Warmblood horses, different reactions were observed to several recombinant *C. nubeculosus* and *C. obsoletus* antigens, where generally *C. obsoletus* allergens best differentiated between cases and controls [29]. Hardly any *C. nubeculosus* and *C. sonorensis* were found feeding of the horses in the Netherlands [57]. Correspondingly, the observed IgE levels against *C. nubeculosus* and *C. sonorensis* allergens were lower when compared to levels against *C. obsoletus* [26]. The use of different allergens might have contributed to the differences in results that were obtained in Belgian Warmblood horses as compared to Shetland ponies and Icelandic horses.

While using allergen-specific IgE levels against *Culicoides* spp. in the serum of horses as phenotype for a GWA analysis has allowed for the identification of several new genomic regions of interest. Previous studies used lower density SNP arrays and/or a case-control GWA study design, and they were only able to explain a small part of the IBH heritability [14,15,16,17,18,58]. LD in most breeds declines rapidly, reaching a background level in only 1 or 2 Mb, which indicates that the use of lower density chips will be unable to detect causal mutations at a distance of 2 Mb or more from the genetic marker [59]. In combination with a lower SNP density being employed in Shetland ponies and Icelandic horses, and the use of other *Culicoides* antigens, this might explain why fewer regions were significantly associated in these breeds as compared to the Belgian Warmblood horse breed [58,59,60]. Indeed, when a subset of SNPs was selected in the Belgian Warmblood horses, which represented the same density and SNPs being tested in Shetland ponies and Icelandic horses, the obtained associations were less significant (Appendix D). Therefore, our results seem to indicate that it might require a higher density array to take full benefit of using these IgE levels as a more objective, independent, and quantitative phenotype.

## 5. Conclusions and Implications

Using a diagnostic ELISA test to assess the IgE levels against *Culicoides* spp. allergens provided a more objective, independent, and quantitative phenotype (as compared to IBH case or control) to be used as input for a GWA study. The GWA analyses employing IgE levels as a phenotype seem to have more power than the GWA analyses that used case-control status as the phenotype. Our GWA results confirmed the complex nature of IBH in horses, affirming several previously identified genomic regions that were associated to IBH and identifying new regions of interest, potentially representing additional or other pathways that are involved in IgE levels as compared to the case-control status. Our findings also seem to indicate that the use of a higher density array might be necessary to fully employ the use of allergen-specific IgE levels. Although our results need to be verified in a larger dataset, the use of allergen-specific IgE levels and the availability of high density SNP genotypes allowed for the identification of new candidate regions that are possibly important in the aetiology of IBH and contributing to our understanding of its biology.

## Figures and Tables

**Table 1:** Number of horses tested for (recombinant) allergens of *Culicoides obsoletus* and *Culicoides nubeculosus* in the Belgian Warmblood horse, Icelandic horse, and Shetland pony breeds.

	Breed		
Allergen	Belgian Warmblood Horse	Icelandic Horse	Shetland Pony
Total	214	127	199
Culo1a ^1,2^	193	−	−
Culo1b ^1,2^	−	127	199
Culo2a ^1,2^	165	−	−
Culo2b ^1,2^	−	127	199
Culo3 ^1^	−	127	199
Culo4 ^1^	−	127	199
Culo5 ^1^	−	127	199
Culo6 ^1^	−	127	199
Culo7 ^1^	−	127	199
Combi1 ^3^	−	127	199
Combi2 ^3^	−	124	163
Culn1 ^4^	142	−	−
Culn3 ^4^	187	−	−
Culn4 ^4^	194	−	−
Culn5 ^4^	123	−	−
Culn7 ^4^	167	−	−
Culn8 ^4^	137	−	−
Culn10 ^4^	157	−	−
WBE ^5^	182	−	−
WBE ^6^	−	124	176
nubTH ^7^	135	−	−

^1^ Seven *C. obsoletus* salivary gland recombinant allergens (Culo1–Culo7) [26,29,31,32,36]. ^2^ Similar nomenclature but different proteins were used: Culo1a (Genbank id JX512273) and Culo2a (Genbank ID JX512274) by Peeters and colleagues [29] and Culo1b (Genbank id KC339671) and Culo2b (Genbank ID KC339672) by van der Meide and colleagues [31]. ^3^ Combi1 contained allergens Culo3, 5, and 7 and Combi2 contained allergens Culo1b, 2b, 5, and 7 [32]. ^4^ Seven *C. nubeculosus* salivary gland recombinant allergens (Culn1, 3–5, 7, 8, 10) [25,29,36,37]. ^5^ WBE = whole body extracts from *C. obsoletus* biting midges, as constructed and tested by Peeters and colleagues [29]. ^6^ WBE = whole body extracts from *C. obsoletus* biting midges, as constructed and tested by van der Meide and colleagues [26]. ^7^ Thorax and head (TH) extracts of *C. nubeculosus* (body removed) [29].

**Figure A1:**
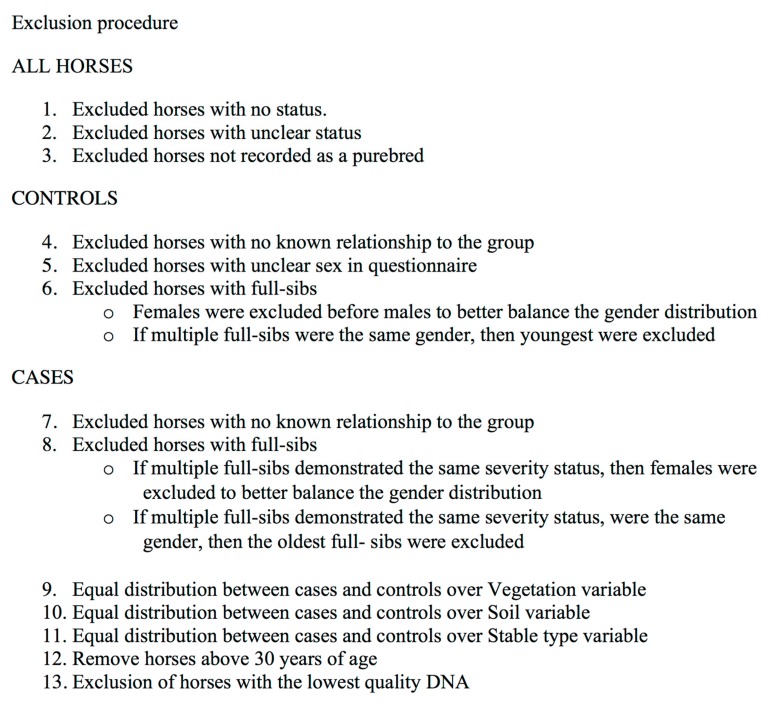
Exclusion protocol used to select Belgian Warmblood horses for genotyping.

**Figure A2:**
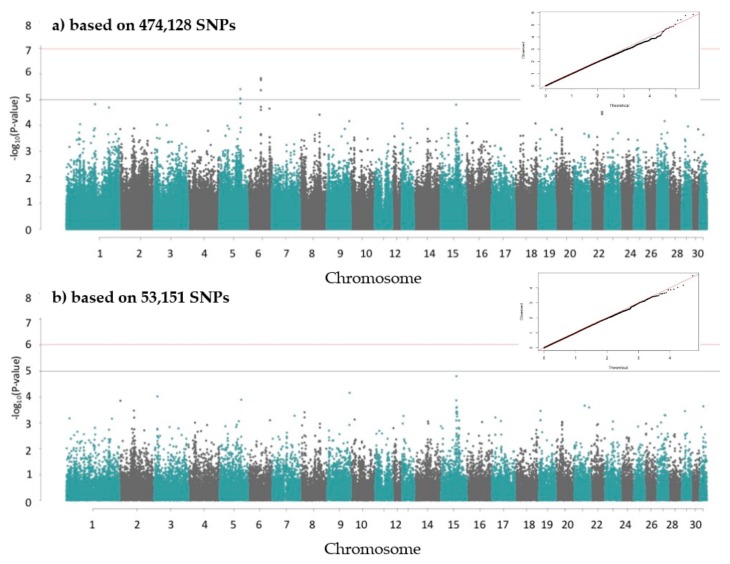
Manhattan plot with position on the genome on the x-axis and significance (−log10(*p*-values)) on the y-axis in the Belgian Warmblood horse population for L_Culn7 using (**a**) all 474,128 SNPs after quality control, or (**b**) a subset of all SNPs representing the density used in the Shetland pony and Icelandic horse populations, being the overlap between the two SNP arrays that were used to genotype the individuals. The red line represents the Bonferonni corrected significance threshold and the black line a nominal significance threshold (*p*-value = 1 × 10^−5^). Q-Q plots indicating that inflation factor lambda was <1, requiring no further adjustment.

## Appendix B

**Table A1:** Descriptive statistics (mean, standard deviation, minimum and maximum) of IgE levels (OD_450nm_ values) and log transformed IgE levels used in the GWA analyses.

	Breed								
	Belgian Warmblood Horse			Icelandic Horse					Shetland Pony
OD_450nm_ Values *Mean (SD) Minimum Maximum*	Cases	Controls	Total	Cases	Controls	Total	Cases	Controls	Total
Culo1a ^2^	0.52 (0.78) 0.001 3.64	0.07 (0.11) 0.003 0.64	0.31 (0.62) 0.0003 3.64	−	−	−	−	−	−
Culo1b ^2^	−	−	−	0.89 (0.43) 0.08 2.18	0.18 (0.27) 0.06 1.80	0.50 (0.38) 0.06 2.18	0.80 (0.44) 0.08 2.21	0.22 (0.12) 0.08 1.02	0.51 (0.35) 0.08 2.21
Culo2a ^2^	0.53 (0.91) 0.0007 3.43	0.08 (0.09) 0.0005 0.43	0.33 (0.72) 0.0005 3.43	−	−	−	−	−	−
Culo2b ^2^	−	−	−	0.42 (0.61) 0.12 2.18	0.15 (0.24) 0.07 1.41	0.27 (0.61) 0.07 2.18	0.44 (0.62) 0.16 2.12	0.17 (0.35) 0.08 2.23	0.31 (0.64) 0.08 2.23
Culo3	−	−	−	1.07 (0.63) 0.13 2.67	0.22 (0.32) 0.07 1.95	0.59 (0.64) 0.07 2.67	1.17 (0.61) 0.10 2.31	0.38 (0.40) 0.07 2.04	0.78 (0.65) 0.07 2.31
Culo4	−	−	−	1.12 (0.37) 0.08 2.06	0.25 (0.28) 0.07 1.81	0.64 (0.34) 0.07 2.06	1.10 (0.44) 0.08 2.07	0.32 (0.22) 0.07 1.39	0.72 (0.38) 0.07 2.07
Culo5	−	−	−	0.37 (0.56) 0.15 2.18	0.15 (0.28) 0.10 1.35	0.24 (0.56) 0.10 2.18	0.48 (0.59) 0.12 2.54	0.20 (0.51) 0.11 2.51	0.34 (0.68) 0.11 2.54
Culo6	−	−	−	0.98 (0.37) 0.07 1.79	0.26 (0.20) 0.07 1.61	0.58 (0.31) 0.07 1.79	1.27 (0.45) 0.07 3.13	0.46 (0.15) 0.07 0.88	0.88 (0.37) 0.07 3.13
Culo7	−	−	−	0.39 (0.56) 0.08 2.26	0.15 (0.43) 0.06 2.81	0.25 (0.51) 0.06 2.81	0.52 (0.61) 0.11 2.62	0.20 (0.40) 0.06 3.04	0.36 (0.55) 0.06 3.08
Combi1 ^3^	−	−	−	1.35 (0.58) 0.28 2.65	0.28 (0.37) 0.07 1.95	0.75 (0.71) 0.07 2.65	1.50 (0.61) 0.11 2.62	0.35 (0.45) 0.08 2.03	0.94 (0.79) 0.08 2.62
Combi2 ^3^	−	−	−	1.24 (0.62) 0.18 2.29	0.31 (0.43) 0.08 1.88	0.72 (0.70) 0.08 2.29	1.44 (0.63) 0.17 2.42	0.41 (0.48) 0.11 2.14	0.93 (0.76) 0.11 2.42
Culn1 ^4^	0.35 (0.73) 0.001 3.56	0.07 (0.12) 0.0003 0.65	0.23 (0.58) 0.0002 3.56	−	−	−	−	−	−
Culn3 ^4^	0.21 (0.40) 0.0003 2.90	0.09 (0.15) 0.0003 1.21	0.15 (0.31) 0.0002 2.90	−	−	−	−	−	−
Culn4 ^4^	0.35 (0.50) 0.002 2.83	0.11 (0.22) 0.0003 1.65	0.24 (0.42) 0.0002 2.83	−	−	−	−	−	−
Culn5 ^4^	0.17 (0.38) 0.001 2.94	0.04 (0.05) 0.0003 0.29	0.11 (0.29) 0.0003 2.95	−	−	−	−	−	−
Culn7 ^4^	0.15 (0.33) 0.005 2.92	0.07 (0.06) 0.0004 0.24	0.12 (0.25) 0.0005 2.92	−	−	−	−	−	−
Culn8 ^4^	0.05 (0.06) 0.0004 0.31	0.04 (0.04) 0.0007 0.22	0.05 (0.05) 0.0004 0.31	−	−	−	−	−	−
Culn10 ^4^	0.13 (0.17) 0.0007 0.84	0.09 (0.12) 0.0003 0.83	0.11 (0.15) 0.0003 0.84	−	−	−	−	−	−
WBE ^5^	0.20 (0.20) 0.0002 1.04	0.09 (0.10) 0.0003 0.67	0.15 (0.17) 0.0002 1.04	−	−	−	−	−	−
WBE ^6^	−	−	−	0.89 (0.49) 0.13 2.08	0.18 (0.21) 0.08 1.71	0.50 (0.51) 0.08 2.08	0.80 (0.43) 0.13 1.92	0.22 (0.12) 0.09 0.59	0.51 (0.44) 0.09 1.92
nubTH ^7^	0.18 (0.18) 0.001 1.02	0.07 (0.08) 0.0006 0.35	0.14 (0.16) 0.0006 1.02	−	−	−	−	−	−
**Log transformed OD_450nm_ values ^1^** *Mean (SD)* *Minimum−maximum*									
L_Culo1a ^2^	1.77 (1.64) −1.29 6.55	3.44 (1.42) 0.44 8.27	2.54 (1.75) −1.29 8.27	−	−	−	−	−	−
L_Culo1b ^2^	−	−	−	−1.27 (0.86) −2.47 0.78	−2.27 (0.56) −2.77 0.59	−1.83 (0.87) −2.77 0.78	−1.19 (0.81) −2.54 0.79	−1.90 (0.43) −2.58 0.02	−1.54 (0.74) −2.58 0.79
L_Culo2a ^2^	1.92 (1.80) −1.23 7.19	3.29 (1.41) 0.83 7.60	2.51 (1.77) −1.23 7.60	−	−	−	−	−	−
L_Culo2b ^2^	−	−	−	−0.19 (0.80) −2.15 0.78	−1.76 (0.61) −2.65 0.34	−1.07 (1.05) −2.65 0.78	−0.05 (0.73) −1.85 0.75	−1.26 (0.70) −2.56 0.80	−0.64 (0.93) −2.56 0.80
L_Culo3	−	−	−	−0.13 (0.79) −2.07 0.98	−1.76 (0.77) −2.72 0.67	−1.04 (1.12) −2.72 0.98	−0.14 (0.79) −2.35 0.84	−1.57 (0.78) −2.71 0.71	0.84 (1.06) −2.71 0.84
L_Culo4	−	−	−	−1.32 (0.73) −2.47 0.72	−2.25 (0.55) −2.70 0.59	−1.84 (0.79) −2.70 0.72	−1.08 (0.82) −2.52 0.73	−1.89 (0.60) −2.64 0.33	−1.48 (0.82) −2.64 0.73
L_Culo5	−	−	−	−0.23 (0.72) −1.90 0.78	−1.64 (0.67) −2.28 0.30	−1.02 (0.98) −2.28 0.78	0.07 (0.66) −2.13 0.93	−1.12 (0.75) −2.21 0.92	−0.51 (0.92) −2.21 0.93
L_Culo6	−	−	−	−1.29 (0.80) −2.60 0.58	−2.17 (0.56) −2.72 0.47	−1.78 (0.81) −2.72 0.58	−0.96 (0.79) −2.64 1.14	−1.79 (0.55) −2.61 −0.13	−1.37 (0.80) −2.64 1.14
L_Culo7	−	−	−	−1.19 (0.98) −2.58 0.81	−2.11 (0.78) −2.74 1.03	−1.71 (0.99) −2.74 1.03	−1.15 (0.99) −2.73 1.12	−2.17 (0.56) −2.88 1.11	−1.65 (0.96) −2.88 1.12
L_Combi1 ^3^	−	−	−	0.19 (0.51) −1.28 0.97	−1.74 (0.83) −2.60 0.67	−0.89 (1.19) −2.60 0.97	0.26 (0.64) −2.16 0.96	−1.48 (0.79) −2.58 0.71	−0.59 (1.13) −2.58 0.96
L_Combi2 ^3^	−	−	−	0.05 (0.62) −1.69 0.83	−2.66 (0.82) −2.49 0.63	−0.90 (1.12) −2.49 0.83	0.20 (0.66) −1.76 0.88	−1.28 (0.77) −2.23 0.76	−0.53 (1.03) −2.23 0.88
L_Culn1 ^4^	2.72 (1.89) −1.27 6.80	3.41 (1.44) 0.43 8.24	3.01 (1.75) −1.27 8.24	−	−	−	−	−	−
L_Culn3 ^4^	2.52 (1.51) −1.07 8.23	3.17 (1.35) −0.19 7.91	2.83 (1.47) −1.07 8.23	−	−	−	−	−	−
L_Culn4 ^4^	1.90 (1.48) −1.04 6.35	3.14 (1.49) −0.50 8.29	2.46 (1.61) −1.04 8.29	−	−	−	−	−	−
L_Culn5 ^4^	2.97 (1.66) −1.08 6.95	3.67 (1.27) 1.23 8.04	3.28 (1.53) −1.08 8.04	−	−	−	−	−	−
L_Culn7 ^4^	2.62 (1.20) −1.07 5.40	3.28 (1.29) 1.41 7.63	2.91 (1.28) −1.07 7.63	−	−	−	−	−	−
L_Culn8 ^4^	3.63 (1.44) 1.18 7.71	3.95 (1.32) 1.50 7.23	3.78 (1.39) 1.18 7.71	−	−	−	−	−	−
L_Culn10 ^4^	3.10 (1.76) 0.18 7.23	3.11 (1.42) 0.19 8.29	3.10 (1.62) 0.18 8.29	−	−	−	−	−	−
L_WBE ^5^	2.28 (1.50) −0.04 8.34	3.02 (1.35) 0.40 8.27	2.61 (1.48) −0.04 8.34	−	−	−	−	−	−
L_WBE ^6^	−	−	−	−0.30 (0.67) −2.06 0.73	−1.94 (0.53) −2.52 0.54	−1.22 (1.01) −2.52 0.73	−0.40 (0.64) −2.01 0.65	−1.67 (0.49) −2.37 −0.52	−1.02 (0.85) −2.37 0.65
L_nubTH ^7^	2.19 (1.17) −0.02 6.70	3.53 (1.53) 1.06 7.40	2.72 (1.48) −1.23 7.60	−	−	−	−	−	−

^1^ IgE levels (OD_450nm_ values) against *Culicoides* spp. were log transformed using the natural logarithm in Icelandic horses and Shetland ponies and using the negative natural logarithm (−ln(OD_450nm_ values)) in Belgian Warmblood horses. ^2^ Similar nomenclature but different proteins were used: Culo1a (Genbank id JX512273) and Culo2a (Genbank ID JX512274) by Peeters and colleagues [29] and Culo1b (Genbank id KC339671) and Culo2b (Genbank ID KC339672) by van der Meide and colleagues [31]. ^3^ Combi1 contained allergens Culo3, 5, and 7 and Combi2 contained allergens Culo1b, 2b, 5, and 7 [32]. ^4^ Seven *C. nubeculosus* salivary gland recombinant allergens (Culn1, 3–5, 7, 8, 10) [25,29,36,37]. ^5^ WBE = whole body extracts from *C. obsoletus* biting midges, as constructed and tested by Peeters and colleagues [29]. ^6^ WBE = whole body extracts from *C. obsoletus* biting midges, as constructed and tested by van der Meide and colleagues [26]. ^7^ Thorax and head (TH) extracts of *C. nubeculosus* (body removed) [29].

## Appendix C

**Table A2:** SNPs most significantly associated with IgE levels against various Culicoides antigens in Belgian Warmblood horses, Icelandic horses and Shetland ponies. As none of the SNPs passed the Bonferroni corrected significance threshold (1.01 × 10^−6^ in Shetland ponies, 9.50 × 10^−7^ in Icelandic horses, and 1.05 × 10^−7^ in Belgian Warmblood horses), per breed the 50 most significantly associated SNPs are presented, including location, allele frequency, allele substitution effect and *p*-value.

SNP	Breed	Chr ^1^	Pos ^2^	IgE level ^3^	A1 ^4^	A2 ^4^	Freq A2 ^5^	β^SE 6^	Pc1df ^7^
BIEC2_27204	Icelandic horse	1	62,517,648	L_Culo6	B	A	0.03	1.339^0.318^	2.81 × 10^−5^
BIEC2_27204	Icelandic horse	1	62,517,648	L_Culo4	B	A	0.03	1.213^0.308^	1.06 × 10^−4^
BIEC2_28234	Icelandic horse	1	64,386,608	L_Culo6	A	B	0.05	0.980^0.239^	4.64 × 10^−5^
BIEC2_37747	Shetland pony	1	81,341,895	L_Culo4	B	A	0.14	0.451^0.122^	2.25 × 10^−4^
AX-103503964	Belgian Warmblood horse	1	100,404,573	L_Culn7	A	G	0.01	2.766^0.639^	1.50 × 10^−5^
BIEC2_45214	Icelandic horse	1	107,931,611	L_Culo4	A	B	0.21	0.491^0.120^	5.91 × 10^−5^
BIEC2_45649	Icelandic horse	1	109,071,746	L_Culo5	B	A	0.26	0.569^0.144^	8.00 × 10^−5^
BIEC2_468481	Icelandic horse	1	111,803,287	L_Culo4	A	B	0.08	0.809^0.178^	7.44 × 10^−6^
BIEC2_468521	Icelandic horse	1	111,806,815	L_Culo4	A	B	0.08	0.809^0.178^	7.44 × 10^−6^
AX-103346536	Belgian Warmblood horse	1	155,846,866	L_WBE	A	G	0.02	2.704^0.577^	3.98 × 10^−6^
BIEC2_458516	Icelandic horse	2	16,510,393	L_Culo4	B	A	0.04	1.112^0.272^	5.09 × 10^−5^
BIEC2_458516	Icelandic horse	2	16,510,393	L_WBE	B	A	0.04	1.369^0.341^	6.97 × 10^−5^
BIEC2_477913	Icelandic horse	2	52,087,373	L_Culo4	B	A	0.06	0.935^0.232^	7.27 × 10^−5^
BIEC2_477926	Icelandic horse	2	52,126,031	L_Culo4	A	B	0.06	0.935^0.232^	7.27 × 10^−5^
BIEC2_477984	Icelandic horse	2	52,282,449	L_Culo4	B	A	0.04	1.053^0.257^	5.59 × 10^−5^
BIEC2_478173	Shetland pony	2	53,366,587	L_Culo7	B	A	0.23	0.440^0.111^	9.34 × 10^−5^
BIEC2-478967	Icelandic horse	2	55,097,850	L_Culo6	A	B	0.01	2.160^0.475^	5.99 × 10^−6^
BIEC2_478970	Icelandic horse	2	55,101,939	L_Culo6	B	A	0.01	2.160^0.475^	5.99 × 10^−6^
AX-104248441	Belgian Warmblood horse	2	57,180,618	L_WBE	G	A	0.30	0.746^0.171^	1.80 × 10^−5^
AX-104413239	Belgian Warmblood horse	2	57,795,030	L_WBE	G	A	0.30	0.759^0.168^	8.58 × 10^−6^
BIEC2_483351	Icelandic horse	2	63,034,801	L_Culo1b	A	B	0.04	1.172^0.304^	1.18 × 10^−4^
BIEC2_484391	Shetland pony	2	64,289,972	L_Culo7	B	A	0.04	0.914^0.240^	1.89 × 10^−4^
BIEC2_484575	Shetland pony	2	65,851,405	L_Culo7	A	B	0.04	0.974^0.246^	1.00 × 10^−4^
BIEC2_491244	Shetland pony	2	78,068,427	L_Culo6	A	B	0.37	0.329^0.082^	6.10 × 10^−5^
BIEC2_522114	Shetland pony	2	89,890,319	L_Culo7	B	A	0.21	0.465^0.118^	1.07 × 10^−4^
TBIEC2_523156	Shetland pony	2	91,300,136	L_Culo1b	B	A	0.13	0.389^0.104^	1.81 × 10^−4^
AX-104656854	Belgian Warmblood horse	2	107,184,258	L_Culo1a	G	A	0.01	3.847^0.889^	1.50 × 10^−5^
AX-104651894	Belgian Warmblood horse	3	15,403,585	L_Culn5	C	T	0.40	−0.919^0.210^	1.78 × 10^−5^
BIEC2_773441	Icelandic horse	3	17,214,985	L_Combi1	A	B	0.12	0.978^0.252^	1.04 × 10^−4^
BIEC2_813132	Shetland pony	3	17,751,670	L_Culo1b	B	A	0.10	0.487^0.132^	2.37 × 10^−4^
BIEC2_782301	Shetland pony	3	54,836,579	L_Culo3	B	A	0.47	0.404^0.110^	2.42 × 10^−4^
BIEC2-791649	Shetland pony	3	71,803,910	L_Culo4	A	B	0.02	1.375^0.345^	7.18 × 10^−5^
BIEC2-791649	Shetland pony	3	71,803,910	L_WBE	A	B	0.02	1.447^0.366^	7.77 × 10^−5^
BIEC2_794498	Icelandic horse	3	78,402,173	L_Culo7	B	A	0.38	0.532^0.131^	5.15 × 10^−5^
BIEC2_794498	Icelandic horse	3	78,402,173	L_Culo5	B	A	0.38	0.544^0.137^	7.46 × 10^−5^
BIEC2_794499	Icelandic horse	3	78,402,249	L_Culo7	A	B	0.38	0.532^0.131^	5.15 × 10^−5^
BIEC2_794499	Icelandic horse	3	78,402,249	L_Culo5	A	B	0.38	0.544^0.137^	7.46 × 10^−5^
BIEC2_844842	Shetland pony	4	4,540,728	L_Culo6	B	A	0.06	0.745^0.168^	9.48 × 10^−6^
BIEC2_844842	Shetland pony	4	4,540,728	L_Culo4	B	A	0.06	0.708^0.173^	4.60 × 10^−5^
BIEC2_844876	Shetland pony	4	4,590,809	L_Culo6	B	A	0.09	0.504^0.137^	2.43 × 10^−4^
AX-103506369	Belgian Warmblood horse	4	44,741,394	L_Culn3	T	C	0.02	−3.338^0.755^	1.00 × 10^−5^
AX-103506369	Belgian Warmblood horse	4	44,741,394	L_Culn1	T	C	0.02	−3.338^0.755^	1.00 × 10^−5^
AX-103506369	Belgian Warmblood horse	4	44,741,394	L_Culn4	T	C	0.02	−3.338^0.755^	1.00 × 10^−5^
AX-103103078	Belgian Warmblood horse	4	44,929,049	L_Culn3	C	T	0.02	−3.333^0.755^	1.00 × 10^−5^
AX-103103078	Belgian Warmblood horse	4	44,929,049	L_Culn1	C	T	0.02	−3.333^0.755^	1.00 × 10^−5^
AX-103103078	Belgian Warmblood horse	4	44,929,049	L_Culn4	C	T	0.02	−3.333^0.755^	1.00 × 10^−5^
BIEC2-898668	Icelandic horse	5	25,944,960	L_Culo3	B	A	0.14	0.842^0.214^	8.50 × 10^−5^
BIEC2_898714	Icelandic horse	5	26,215,264	L_Culo5	B	A	0.35	−0.576^0.150^	1.20 × 10^−4^
AX-103271642	Belgian Warmblood horse	5	74,007,505	L_Culn7	A	G	0.23	−0.725^0.163^	9.08 × 10^−6^
AX-104561711	Belgian Warmblood horse	5	74,007,642	L_Culn7	G	A	0.23	−0.755^0.163^	3.89 × 10^−6^
AX-104085455	Belgian Warmblood horse	5	74,013,902	L_Culn7	C	T	0.23	−0.712^0.164^	1.41 × 10^−5^
AX-103834157	Belgian Warmblood horse	5	78,269,939	L_Culn5	A	C	0.30	−1.012^0.229^	1.40 × 10^−5^
BIEC2_933647	Shetland pony	5	98,392,335	L_Culo7	B	A	0.03	1.157^0.311^	2.56 × 10^−4^
BIEC2_935332	Shetland pony	6	1,212,183	L_Culo4	A	B	0.10	0.535^0.138^	1.18 × 10^−4^
BIEC2_983237	Shetland pony	6	1,328,824	L_Culo4	B	A	0.10	0.533^0.138^	1.26 × 10^−4^
BIEC2_938056	Icelandic horse	6	6,834,306	L_Culo1b	A	B	0.19	0.632^0.140^	6.24 × 10^−6^
BIEC2-941245	Shetland pony	6	13,162,030	L_Culo4	B	A	0.11	0.574^0.134^	2.10 × 10^−5^
BIEC2_990454	Shetland pony	6	15,765,010	L_Culo7	B	A	0.01	1.949^0.437^	1.18 × 10^−5^
BIEC2_990454	Shetland pony	6	15,765,010	L_Culo6	B	A	0.01	1.405^0.367^	1.27 × 10^−4^
AX-104276231	Belgian Warmblood horse	6	44,759,822	L_Culn8	C	T	0.06	1.815^0.406^	7.85 × 10^−6^
AX-104807788	Belgian Warmblood horse	6	45,879,727	L_Culn7	A	G	0.07	1.284^0.279^	4.27 × 10^−6^
AX-103196232	Belgian Warmblood horse	6	45,975,297	L_Culn7	G	A	0.10	1.037^0.242^	1.90 × 10^−5^
AX-103555139	Belgian Warmblood horse	6	46,095,918	L_Culn7	T	C	0.14	0.997^0.208^	1.72 × 10^−6^
AX-103105514	Belgian Warmblood horse	6	46,125,756	L_Culn7	G	A	0.14	1.011^0.210^	1.46 × 10^−6^
AX-103811007	Belgian Warmblood horse	6	59,819,569	L_WBE	T	C	0.25	0.840^0.193^	1.78 × 10^−5^
BIEC2_1017806	Icelandic horse	6	73,076,296	L_Culo3	B	A	0.30	0.571^0.140^	4.63 × 10^−5^
AX-103340585	Belgian Warmblood horse	6	79,707,409	L_WBE	T	C	0.01	2.793^0.621^	9.41 × 10^−6^
BIEC2_984144	Icelandic horse	7	19,118,876	L_Culo2b	A	B	0.47	0.535^0.133^	5.64 × 10^−5^
BIEC2_984146	Icelandic horse	7	19,118,997	L_Culo2b	A	B	0.47	0.535^0.133^	5.64 × 10^−5^
BIEC2_1002598	Icelandic horse	7	56,761,597	L_Culo1b	A	B	0.40	−0.450^0.117^	1.21 × 10^−4^
AX-104003295	Belgian Warmblood horse	7	82,435,491	L_WBE	T	C	0.07	1.469^0.305^	2.09 × 10^−6^
BIEC2-1010453	Shetland pony	7	85,717,480	L_Culo6	B	A	0.55	0.298^0.079^	1.68 × 10^−4^
BIEC2-1010454	Shetland pony	7	85,717,499	L_Culo6	A	B	0.55	0.298^0.079^	1.68 × 10^−4^
BIEC2_10104552	Shetland pony	7	85,717,606	L_Culo6	A	B	0.55	0.298^0.079^	1.68 × 10^−4^
BIEC2_1096840	Icelandic horse	8	27,964,452	L_Culo7	B	A	0.07	1.070^0.242^	1.04 × 10^−5^
AX-103533308	Belgian Warmblood horse	8	64,692,596	L_WBE	A	G	0.07	1.405^0.314^	1.08 × 10^−5^
AX-103391816	Belgian Warmblood horse	8	64,784,788	L_WBE	C	T	0.06	1.490^0.331^	9.18 × 10^−6^
BIEC2_1063702	Icelandic horse	8	79,554,471	L_Culo4	B	A	0.02	1.307^0.336^	1.28 × 10^−4^
BIEC2_1064164	Shetland pony	8	82,054,284	L_Culo6	B	A	0.03	−0.843^0.230^	2.41 × 10^−4^
BIEC2_1125196	Shetland pony	8	92,699,636	L_Culo4	A	B	0.09	0.522^0.139^	1.84 × 10^−4^
BIEC2_1141819	Shetland pony	9	27,136,208	L_Culo1b	B	A	0.02	1.103^0.282^	8.98 × 10^−5^
AX-104628650	Belgian Warmblood horse	10	19,284,909	L_Culn5	G	A	0.08	1.627^0.353^	5.88 × 10^−6^
BIEC2_108460	Icelandic horse	10	21,301,438	L_Culo4	B	A	0.73	0.453^0.113^	8.24 × 10^−5^
BIEC2_122837	Shetland pony	10	51,100,177	L_Culo4	A	B	0.08	0.559^0.153^	2.68 × 10^−4^
BIEC2_129582	Shetland pony	10	64,203,886	L_Culo7	B	A	0.01	1.911^0.435^	1.57 × 10^−5^
TBIEC2_136550	Shetland pony	10	64,532,326	L_Culo7	B	A	0.26	0.485^0.110^	1.55 × 10^−5^
BIEC2_131860	Icelandic horse	10	76,735,207	L_Culo3	B	A	0.03	1.784^0.451^	7.54 × 10^−5^
BIEC2_152809	Icelandic horse	11	40,721,405	L_WBE	A	B	0.47	−0.562^0.137^	4.95 × 10^−5^
AX-103887059	Belgian Warmblood horse	11	43,666,485	L_Culn3	C	T	0.07	−1.795^0.415^	1.55 × 10^−5^
AX-103887059	Belgian Warmblood horse	11	43,666,485	L_Culn1	C	T	0.07	−1.795^0.415^	1.55 × 10^−5^
AX-103887059	Belgian Warmblood horse	11	43,666,485	L_Culn4	C	T	0.07	−1.795^0.415^	1.55 × 10^−5^
AX-103378324	Belgian Warmblood horse	12	7,216,566	L_Culo2a	T	C	0.02	3.183^0.703^	5.90 × 10^−6^
AX-103538080	Belgian Warmblood horse	12	19,588,916	L_Culo1a	T	G	0.12	−1.200^0.278^	1.60 × 10^−5^
AX-104700154	Belgian Warmblood horse	13	2,214,862	L_Culn8	G	A	0.48	−0.844^0.183^	4.16 × 10^−6^
AX-104619436	Belgian Warmblood horse	13	11,360,389	L_WBE	C	T	0.28	0.803^0.172^	4.18 × 10^−6^
BIEC2_223584	Icelandic horse	13	17,801,684	L_Culo5	A	B	0.30	0.543^0.137^	7.07 × 10^−5^
BIEC2_223584	Icelandic horse	13	17,801,684	L_Culo6	A	B	0.30	0.458^0.116^	9.00 × 10^−5^
BIEC2-239709	Shetland pony	14	4,274,260	L_Combi2	B	A	0.38	0.527^0.136^	1.03 × 10^−4^
BIEC2_245596	Shetland pony	14	13,580,552	L_Culo4	A	B	0.19	−0.420^0.104^	5.28 × 10^−5^
BIEC2_259420	Shetland pony	14	54,397,113	L_Culo7	B	A	0.01	1.873^0.482^	1.33 × 10^−4^
AX-104663195	Belgian Warmblood horse	14	55,773,239	L_Culn8	C	T	0.02	3.097^0.687^	6.64 × 10^−6^
BIEC2_262621	Icelandic horse	14	67,999,643	L_Combi2	B	A	0.49	−0.648^0.158^	3.99 × 10^−5^
BIEC2_262673	Icelandic horse	14	68,068,555	L_Combi2	A	B	0.53	−0.620^0.152^	4.67 × 10^−5^
BIEC2-289295	Icelandic horse	15	14,642,890	L_Combi1	B	A	0.10	−1.004^0.241^	3.00 × 10^−5^
BIEC2-289295	Icelandic horse	15	14,642,890	L_Combi2	B	A	0.10	−0.923^0.229^	5.72 × 10^−5^
BIEC2_289301	Icelandic horse	15	14,647,231	L_Combi1	A	B	0.10	−1.004^0.241^	3.00 × 10^−5^
BIEC2_289301	Icelandic horse	15	14,647,231	L_Combi2	A	B	0.10	−0.923^0.229^	5.72 × 10^−5^
BIEC2-294189	Icelandic horse	15	21,210,210	L_Culo2b	B	A	0.26	0.651^0.191^	5.23 × 10^−5^
AX-103998137	Belgian Warmblood horse	15	31,741,601	L_Culn8	A	C	0.44	0.763^0.174^	1.22 × 10^−5^
AX-104442678	Belgian Warmblood horse	15	54,538,291	L_Culn7	G	A	0.03	−1.641^0.380^	1.57 × 10^−5^
BIEC2_355030	Icelandic horse	16	62,046,387	L_Combi2	A	B	0.19	0.760^0.194^	8.77 × 10^−5^
BIEC2_355030	Icelandic horse	16	62,046,387	L_Culo5	A	B	0.19	0.639^0.166^	1.21 × 10^−4^
BIEC2_373511	Shetland pony	17	19,689,865	L_Culo4	B	A	0.02	1.229^0.319^	1.21 × 10^−4^
BIEC2_395885	Shetland pony	17	45,644,294	L_Culo6	B	A	0.04	−0.744^0.203^	2.42 × 10^−4^
AX-103325608	Belgian Warmblood horse	17	74,894,091	L_WBE	G	A	0.01	3.865^0.864^	1.06 × 10^−5^
AX-103932279	Belgian Warmblood horse	17	75,173,007	L_WBE	T	C	0.01	3.865^0.864^	1.06 × 10^−5^
AX-104268962	Belgian Warmblood horse	17	75,184,430	L_WBE	A	C	0.02	3.449^0.675^	4.92 × 10^−7^
AX-104514365	Belgian Warmblood horse	18	26,032,740	L_Culn5	A	G	0.04	2.170^0.499^	1.90 × 10^−5^
AX-103909004	Belgian Warmblood horse	18	58,215,227	L_Culn10	T	C	0.01	4.244^0.965^	1.08 × 10^−5^
AX-104652798	Belgian Warmblood horse	18	58,248,171	L_Culn10	G	T	0.01	4.250^0.965^	1.05 × 10^−5^
TBIEC2_461038	Icelandic horse	19	39,283,640	L_Culo7	B	A	0.35	−0.529^0.132^	6.43 × 10^−5^
BIEC2_526197	Shetland pony	20	26,221,179	L_Culo7	B	A	0.11	0.679^0.158^	2.31 × 10^−5^
UKUL3411	Icelandic horse	20	32,902,939	L_Culo4	A	B	0.16	−0.642^0.155^	4.89 × 10^−5^
BIEC2_558521	Shetland pony	20	34,924,555	L_Culo7	A	B	0.59	−0.385^0.098^	1.23 × 10^−4^
UKUL3474	Shetland pony	20	35,643,200	L_WBE	B	A	0.53	−0.416^0.096^	1.33 × 10^−5^
UKUL3474	Shetland pony	20	35,643,200	L_Culo7	B	A	0.53	−0.371^0.098^	2.10 × 10^−4^
UKUL3477	Shetland pony	20	35,692,652	L_WBE	A	B	0.45	0.407^0.096^	2.36 × 10^−5^
UKUL3477	Shetland pony	20	35,692,652	L_Culo7	A	B	0.45	0.382^0.099^	1.51 × 10^−4^
BIEC2_532826	Shetland pony	20	42,983,718	L_Culo1b	B	A	0.45	0.292^0.075^	9.39 × 10^−5^
BIEC2_538781	Shetland pony	20	51,509,431	L_Culo1b	A	B	0.27	−0.337^0.087^	1.01 × 10^−4^
AX-104089679	Belgian Warmblood horse	20	52,011,107	L_nubTH	C	T	0.02	3.785^0.884^	1.87 × 10^−5^
BIEC2_560852	Shetland pony	21	30,022,509	L_Culo7	B	A	0.37	0.372^0.099^	2.38 × 10^−4^
BIEC2_560854	Shetland pony	21	30,022,801	L_Culo7	A	B	0.37	0.372^0.099^	2.38 × 10^−4^
BIEC2_560857	Shetland pony	21	30,033,646	L_Culo7	B	A	0.37	0.372^0.099^	2.38 × 10^−4^
BIEC2_561034	Icelandic horse	21	31,044,321	L_Culo6	A	B	0.04	1.069^0.272^	8.97 × 10^−5^
AX-103656067	Belgian Warmblood horse	22	1,680,310	L_WBE	C	T	0.11	1.234^0.258^	2.42 × 10^−6^
BIEC2_577012	Icelandic horse	22	4,630,951	L_Culo7	A	B	0.10	0.845^0.213^	7.31 × 10^−5^
AX-104834793	Belgian Warmblood horse	22	41,631,523	L_Culn5	G	A	0.04	2.236^0.487^	6.33 × 10^−6^
BIEC2-600796	Icelandic horse	22	46,231,600	L_Culo4	A	B	0.33	0.465^0.114^	6.49 × 10^−5^
BIEC2_626250	Shetland pony	23	48,348,163	L_Culo4	A	B	0.28	0.327^0.089^	2.47 × 10^−4^
BIEC2_626256	Shetland pony	23	48,348,439	L_Culo7	B	A	0.25	0.423^0.114^	2.58 × 10^−4^
BIEC2_658221	Shetland pony	23	48,411,716	L_Culo6	A	B	0.32	−0.340^0.092^	2.33 × 10^−4^
BIEC2_658438	Shetland pony	23	49,092,678	L_Culo6	A	B	0.31	−0.353^0.093^	1.38 × 10^−4^
BIEC2_706297	Shetland pony	25	35,644,297	L_Culo1b	B	A	0.13	−0.404^0.109^	2.01 × 10^−4^
AX-103278700	Belgian Warmblood horse	26	7,825,717	L_Culo1a	T	C	0.22	−1.001^0.233^	1.78 × 10^−5^
AX-103417398	Belgian Warmblood horse	26	9,338,672	L_Culo2a	A	G	0.09	−1.831^0.383^	1.72 × 10^−6^
BIEC2_686588	Icelandic horse	26	16,094,903	L_Culo4	B	A	0.20	0.534^0.136^	1.15 × 10^−4^
BIEC2-692781	Icelandic horse	26	30,306,210	L_Culo3	A	B	0.51	−0.613^0.148^	3.44 × 10^−5^
AX-104737542	Belgian Warmblood horse	27	23,911,229	L_Culn5	T	C	0.46	0.835^0.181^	5.88 × 10^−6^
AX-103948838	Belgian Warmblood horse	29	12,952,739	L_Culn10	T	C	0.06	1.728^0.400^	1.53 × 10^−5^

^1^ Chr = chromosome. ^2^ Pos = position in base pairs. ^3^ IgE levels against *Culicoides* spp. were log transformed. ^4^ A1 = allele 1; A2 = allele 2. ^5^ Freq A2 = frequency of allele 2. ^6^ β^SE^ = allele substitution effect using “*mmscore*” function, with standard error in superscript. ^7^ Pc1df = *p*-value corrected for inflation due to stratification, based on 1 degree of freedom, using “*mmscore*” function, corrected for substructure by use of the assigned cluster.

## Appendix F

**Table A3:** Comprehensive list of regions associated with antigen-specific IgE levels identified in any of the three investigated breeds, along with previously identified genomic regions through a case-control approach in proximity.

Study	Breed	Chr ^1^	Pos (Mb) ^2^
Current study	Icelandic horse	1	62
Current study	Icelandic horse	1	64
Current study	Shetland pony	1	81
Current study	Belgian Warmblood horse	1	100
Velie and colleagues [18]	Exmoor pony	1	100
Current study	Icelandic horse	1	107
Current study	Icelandic horse	1	109
Current study	Icelandic horse	1	111
Schurink and colleagues [15]	Shetland pony	1	116
Current study	Belgian Warmblood horse	1	155
Current study	Icelandic horse	2	16
Current study	Icelandic horse	2	52
Current study	Shetland pony	2	53
Current study	Icelandic horse	2	55
Current study	Belgian Warmblood horse	2	57
Current study	Icelandic horse	2	63
Current study	Shetland pony	2	64
Current study	Shetland pony	2	65
Current study	Shetland pony	2	78
Current study	Shetland pony	2	89
Current study	Shetland pony	2	91
Schurink and colleagues [16]	Friesian horse	2	105
Current study	Belgian Warmblood horse	2	107
Velie and colleagues [18]	Exmoor pony	2	116
Schurink and colleagues [14]	Shetland pony	3	8
Schurink and colleagues [15]	Shetland pony	3	8
Current study	Belgian Warmblood horse	3	15
Current study	Icelandic horse	3	17
Current study	Shetland pony	3	17
Schurink and colleagues [14]	Shetland pony	3	17
Schurink and colleagues [14]	Shetland pony	3	50–51
Current study	Shetland pony	3	54
Schurink and colleagues [15]	Shetland pony	3	64
Current study	Shetland pony	3	71
Current study	Icelandic horse	3	78
Shrestha and colleagues [17]	Icelandic horse	3	87
Velie and colleagues [18]	Exmoor pony	4	1
Current study	Shetland pony	4	4
Schurink and colleagues [14]	Icelandic horse	4	43
Current study	Belgian Warmblood horse	4	44
Current study	Icelandic horse	5	25–26
Schurink and colleagues [14]	Icelandic horse	5	26
Shrestha and colleagues [17]	Icelandic horse	5	71
Current study	Belgian Warmblood horse	5	74
Current study	Belgian Warmblood horse	5	78
Current study	Shetland pony	5	98
Current study	Shetland pony	6	1
Schurink and colleagues [14]	Icelandic horse	6	6
Current study	Icelandic horse	6	6
Current study	Shetland pony	6	13
Current study	Shetland pony	6	15
Current study	Belgian Warmblood horse	6	44–46
Schurink and colleagues [15]	Shetland pony	6	47
Shrestha and colleagues [17]	Icelandic horse	6	48
Velie and colleagues [18]	Exmoor pony	6	55
Current study	Belgian Warmblood horse	6	59
Current study	Icelandic horse	6	73
Current study	Belgian Warmblood horse	6	79
Schurink and colleagues [15]	Shetland pony	7	13
Current study	Icelandic horse	7	19
Schurink and colleagues [14]	Icelandic horse	7	55
Current study	Icelandic horse	7	56
Shrestha and colleagues [17]	Icelandic horse	7	57
Schurink and colleagues [15]	Shetland pony	7	61
Current study	Belgian Warmblood horse	7	82
Current study	Shetland pony	7	85
Schurink and colleagues [14]	Shetland pony	7	85
Current study	Icelandic horse	8	27
Velie and colleagues [18]	Exmoor pony	8	62
Schurink and colleagues [14]	Shetland pony	8	63
Current study	Belgian Warmblood horse	8	64
Schurink and colleagues [15]	Shetland pony	8	70
Velie and colleagues [18]	Exmoor pony	8	70–71
Velie and colleagues [18]	Exmoor pony	8	78–79
Current study	Icelandic horse	8	79
Current study	Shetland pony	8	82
Current study	Shetland pony	8	92
Velie and colleagues [18]	Exmoor pony	9	26
Current study	Shetland pony	9	27
Schurink and colleagues [16]	Friesian horse	10	12–13
Shrestha and colleagues [17]	Icelandic horse	10	19
Current study	Belgian Warmblood horse	10	19
Current study	Icelandic horse	10	21
Shrestha and colleagues [17]	Icelandic horse	10	30
Shrestha and colleagues [17]	Icelandic horse	10	35
Shrestha and colleagues [17]	Icelandic horse	10	41
Shrestha and colleagues [17]	Icelandic horse	10	49
Current study	Shetland pony	10	51
Current study	Shetland pony	10	64
Current study	Icelandic horse	10	76
Schurink and colleagues [14]	Shetland pony	11	32
Schurink and colleagues [15]	Shetland pony	11	32–33
Current study	Icelandic horse	11	40
Schurink and colleagues [14]	Icelandic horse	11	40
Schurink and colleagues [16]	Friesian horse	11	41
Current study	Belgian Warmblood horse	11	43
Current study	Belgian Warmblood horse	12	7
Schurink and colleagues [16]	Friesian horse	12	19
Current study	Belgian Warmblood horse	12	19
Current study	Belgian Warmblood horse	13	2
Current study	Belgian Warmblood horse	13	11
Current study	Icelandic horse	13	17
Current study	Shetland pony	14	4
Current study	Shetland pony	14	13
Current study	Shetland pony	14	54
Current study	Belgian Warmblood horse	14	55
Schurink and colleagues [16]	Friesian horse	14	64
Current study	Icelandic horse	14	67–68
Current study	Icelandic horse	15	14
Schurink and colleagues [14]	Icelandic horse	15	19–20
Current study	Icelandic horse	15	21
Current study	Belgian Warmblood horse	15	31
Schurink and colleagues [14]	Icelandic horse	15	32–33
Current study	Belgian Warmblood horse	15	54
Velie and colleagues [18]	Exmoor pony	16	58–59
Current study	Icelandic horse	16	62
Velie and colleagues [18]	Exmoor pony	16	62–63
Velie and colleagues [18]	Exmoor pony	16	65
Current study	Shetland pony	17	19
Current study	Shetland pony	17	45
Current study	Belgian Warmblood horse	17	74–75
Schurink and colleagues [14]	Shetland pony	17	75–76
Shrestha and colleagues [17]	Icelandic horse	17	77
Current study	Belgian Warmblood horse	18	26
Shrestha and colleagues [17]	Icelandic horse	18	26
Schurink and colleagues [14]	Icelandic horse	18	32
Current study	Belgian Warmblood horse	18	58
Current study	Icelandic horse	19	39
Schurink and colleagues [16]	Friesian horse	20	19
Schurink and colleagues [16]	Friesian horse	20	24
Schurink and colleagues [16]	Friesian horse	20	26
Current study	Shetland pony	20	26
Velie and colleagues [18]	Exmoor pony	20	29
Schurink and colleagues [14]	Icelandic horse	20	30
Schurink and colleagues [16]	Friesian horse	20	29–30
Schurink and colleagues [16]	Friesian horse	20	30–32
Schurink and colleagues [16]	Friesian horse	20	32
Current study	Shetland pony	20	32
Velie and colleagues [18]	Exmoor pony	20	33
Current study	Shetland pony	20	34–35
Schurink and colleagues [14]	Shetland pony	20	35
Schurink and colleagues [14]	Shetland pony	20	41
Schurink and colleagues [15]	Shetland pony	20	41
Current study	Shetland pony	20	42
Velie and colleagues [18]	Exmoor pony	20	49
Current study	Shetland pony	20	51
Current study	Belgian Warmblood horse	20	52
Schurink and colleagues [16]	Friesian horse	21	10
Current study	Shetland pony	21	30
Current study	Icelandic horse	21	31
Current study	Belgian Warmblood horse	22	1
Current study	Icelandic horse	22	4
Current study	Belgian Warmblood horse	22	41
Current study	Icelandic horse	22	46
Schurink and colleagues [16]	Friesian horse	23	32
Current study	Shetland pony	23	48–49
Current study	Shetland pony	25	35
Schurink and colleagues [16]	Friesian horse	26	1
Shrestha and colleagues [17]	Icelandic horse	26	3
Current study	Belgian Warmblood horse	26	7
Current study	Belgian Warmblood horse	26	9
Velie and colleagues [18]	Exmoor pony	26	14
Current study	Icelandic horse	26	16
Current study	Icelandic horse	26	30
Schurink and colleagues [15]	Shetland pony	27	13
Schurink and colleagues [14]	Shetland pony	27	13
Current study	Belgian Warmblood horse	27	23
Current study	Belgian Warmblood horse	29	12

^1^ Chr = chromosome. ^2^ Pos (Mb) = position in mega base pairs.
